# *Bjerkandera* spp. Pulmonary Infection in Immunocompromised Hosts, Germany

**DOI:** 10.3201/eid3111.250878

**Published:** 2025-11

**Authors:** Rosanne Sprute, Danila Seidel, Katrin Mehler, Zoé Westhues, Sarina K. Butzer, Jannik Stemler, Oliver A. Cornely, Philipp Koehler

**Affiliations:** German Centre for Infection Research, Cologne, Germany (R. Sprute, D. Seidel, Z. Westhues, J. Stemler, O.A. Cornely); University of Cologne Faculty of Medicine and University Hospital Cologne Institute of Translational Research, Cologne Excellence Cluster on Cellular Stress Responses in Aging-Associated Diseases, Cologne (R. Sprute, D. Seidel, Z. Westhues, J. Stemler, O.A. Cornely, P. Koehler); University of Cologne Faculty of Medicine and University Hospital Cologne Department of Internal Medicine, Center for Integrated Oncology Aachen Bonn Cologne Duesseldorf and ECMM Excellence Center for Medical Mycology, Cologne (R. Sprute, D. Seidel, Z. Westhues, J. Stemler, O.A. Cornely, P. Koehler); University of Cologne Faculty of Medicine and University Hospital Cologne Department of Pediatrics, Division of Pediatric Infectious Diseases, Cologne (K. Mehler, S.K. Butzer); University of Cologne Faculty of Medicine and University Hospital Cologne, Department of Pediatrics, Division of Pediatric Oncology and Hematology, Cologne (S.K. Butzer); University of Cologne Faculty of Medicine and University Hospital Cologne, Clinical Trials Centre Cologne (ZKS Köln), Cologne (O.A. Cornely); University of Cologne Faculty of Medicine and University Hospital Cologne, Department of Internal Medicine, Division of Clinical Immunology, Cologne (P. Koehler)

**Keywords:** Bjerkandera, fungi, opportunistic infection, rare mold, fungal pneumonia, invasive fungal disease, mycoses, internal transcribed spacer, antifungal, leukemia, basidiomycetes, Germany

## Abstract

We report 3 cases of probable invasive pulmonary disease caused by *Bjerkandera* spp. fungi in immunocompromised patients in Germany. Accurate identification required internal transcribed spacer sequencing. Response to antifungal treatment varied. Our report underlines the pathogenic potential of *Bjerkandera* spp. and the importance of molecular diagnostics in rare fungal infections.

Common molds such as *Aspergillus* spp. and Mucorales are well-recognized pathogens in immunocompromised patients that cause life-threatening invasive fungal disease (IFD). Other environmental molds are frequently dismissed as contaminants in clinical specimens, yet growing evidence through clinical vigilance and advances in molecular techniques has revealed some as emerging threats in vulnerable populations (*1*–*2*). Many of those fungi are expected to remain unidentified because cultures stay negative without prolonged incubation for those organisms, and accurate identification requires molecular methods. Advanced molecular methods, such as cell-free DNA sequencing, hold promise as diagnostic tools but are not yet routinely available (*3*,*4*).

*Bjerkandera* spp., including *B. adusta* (syn. *Geotrichopsis mycoparasitica*) and *B. fumosa,* are filamentous basidiomycetes, wood-decaying fungi that have been isolated from dead hardwood trees in Europe and South America (*5*). *Bjerkandera* spp. have been linked to chronic cough, allergic bronchopulmonary mycosis, and hypersensitivity pneumonitis in humans (*6*–*8*). In addition, invasive sinonasal fungal disease by *B. adusta* was reported in a patient with uncontrolled type 2 diabetes, confirmed through histopathologic examination (*9*). We describe 3 patients in Germany with pulmonary infection and identification of *Bjerkandera* spp. in respiratory specimen that meet the European Organization for Research and Treatment of Cancer and the Mycoses Study Group Education and Research Consortium (EORTC/MSGERC) criteria for probable IFD, highlighting an emerging association between the basidiomycete and human invasive disease.

## The Study

Patient 1 was a 32-year-old man who received allogeneic hematopoietic stem cell transplant (HSCT) for relapsed mediastinal T-cell lymphoma (Table). Nine months later, he experienced progressive dyspnea. The patient was on prednisolone (100 mg/d) immunosuppressive therapy and did not receive antifungal drugs. Computed tomography (CT) of the chest showed new ground-glass opacitis and nodular consolidations. Culture from bronchoalveolar lavage (BAL) fluid revealed a mold identified as *Bjerkandera* spp. by sequencing the internal transcribed spacer (ITS) 1/2 region in accordance with Clinical and Laboratory Standards Institute guidelines (*10*). We did not perform susceptibility testing because fungal growth on the testing media was insufficient. Results of Mucorales and *Aspergillus*-specific PCR from BAL fluid were negative. Serum was negative for galactomannan antigen. We did not perform BAL galactomannan testing. We identified no other potential causes of infectious diseases by culture, PCR, or serology (Table). We initiated empiric antimicrobial therapy with piperacillin/tazobactam for 2 weeks. The fungus was not considered clinically significant; no antifungal treatment was initiated. Immunosuppression was intensified on suspicion of lung graft-versus-host disease, but the patient’s condition continued to deteriorate. We started antifungal therapy with posaconazole both as prophylaxis and targeted treatment of the probable IFD. Eight days later, we performed another BAL in which no fungus or other infectious agent was detected. The patient died shortly afterward from respiratory failure. No autopsy was performed.

**Table:** Characteristics of patients with probable invasive pulmonary disease by *Bjerkandera* spp., Germany*

Patient no.	Age, y	Time of diagnosis and department	Underlying condition and treatment	Antifungal prophylaxis	Radiology	Microbiology	Antifungal Treatment	Outcome
1	32	May 2017, inpatient hematology unit	Relapsed mediastinal T-cell lymphoma; allogeneic HSCT	None	Nodular infiltrates, ground-glass opacities	Mold culture from BAL fluid, *Bjerkandera* spp. identified by ITS sequencing†	Posaconazole	Deceased
2	82	Oct 2022, outpatient hematology department	AML, functional neutropenia; hydroxyurea	None	Nodular infiltrates, cavitary lesion	Mold culture from BAL fluid, *B. adusta* or *B. fumosa* identified by ITS sequencing‡	Voriconazole, isavuconazole	Unknown
3	4	Nov 2022, inpatient pediatric hematology unit	AML; HAM regimen	Micafungin	Nodular infiltrates, ground-glass opacities, cavitary lesion	Mold culture from tracheal aspiration, *B. adusta* or *B. fumosa* identified by ITS sequencing§	Voriconazole, liposomal AmB	Alive, secondary prophylaxis with voriconazole

*Microbiologic analyses were performed at the Institute of Medical Microbiology, Immunology, and Hygiene, University Hospital Cologne, Cologne, Germany. AmB, amphotericin B; AML, acute myeloid leukemia; BAL, bronchoalveolar lavage; HAM, high-dose cytarabine with mitoxantrone; HSCT, hematopoietic stem cell transplant, ITS, internal transcribed spacer. †Other causes of infectious disease were ruled out by negative blood cultures; negative *Legionella* antigen from urine; negative culture from BAL including *Mycobacteria* and *Actinomyces* culture; negative results for *M. tuberculosis* complex, *Chlamydia pneumoniae, Mycoplasma pneumoniae, Legionella pneumophilia*, *Aspergillus* spp., Mucorales, *Pneumocystis jirovecii*, and *Toxoplasma gondii* by PCR from BAL; negative respiratory virus panel from BAL; and negative results on throat swab samples for viruses including influenza A, influenza B, parainfluenza, adenovirus, metapneumovirus, coronavirus, respiratory syncytial virus, rhinovirus, bocavirus, and enterovirus. There was no serologic evidence for *C. pneumoniae* or *M. pneumoniae* infection, no active hepatitis A–E, and negative results for cytomegalovirus, herpes simplex virus, varicella zoster virus, and parvovirus PCR from EDTA blood. We identified Epstein-Barr virus copies 14 IU/mL from EDTA blood, in control virus that was negative by PCR. ‡Other causes of infectious disease were ruled out by negative culture from BAL, including *Mycobacteria* and *Actinomyces* culture; negative results for *M. tuberculosis* complex, *C*.* pneumoniae, M. pneumoniae*, *L. pneumophilia, Aspergillus* spp., Mucorales, *Pneumocystis jirovecii*, and *Toxoplasma gondii* by PCR from BAL; negative influenza A, influenza B, parainfluenza, adenovirus, metapneumovirus, coronavirus, respiratory syncytial virus, rhinovirus, bocavirus, and enterovirus from BAL and throat swab samples; and negative *Legionella* and pneumococcal antigen from urine. §Other causes of infectious disease were ruled out by negative blood cultures and urine cultures; negative culture from tracheal aspiration; negative *C. pneumoniae, L. pneumophilia*, *M. pneumoniae*, *Aspergillus* spp., Mucorales, *Pneumocystis carinii*, and *Toxoplasma gondii* by PCR from tracheal aspiration; and negative cytomegalovirus, human herpesvirus 6A, Epstein-Barr virus, influenza A, influenza B, parainfluenza, adenovirus, metapneumovirus, coronavirus, respiratory syncytial virus, rhinovirus, bocavirus, and enterovirus by PCR from tracheal aspiration.

**Figure 1:**
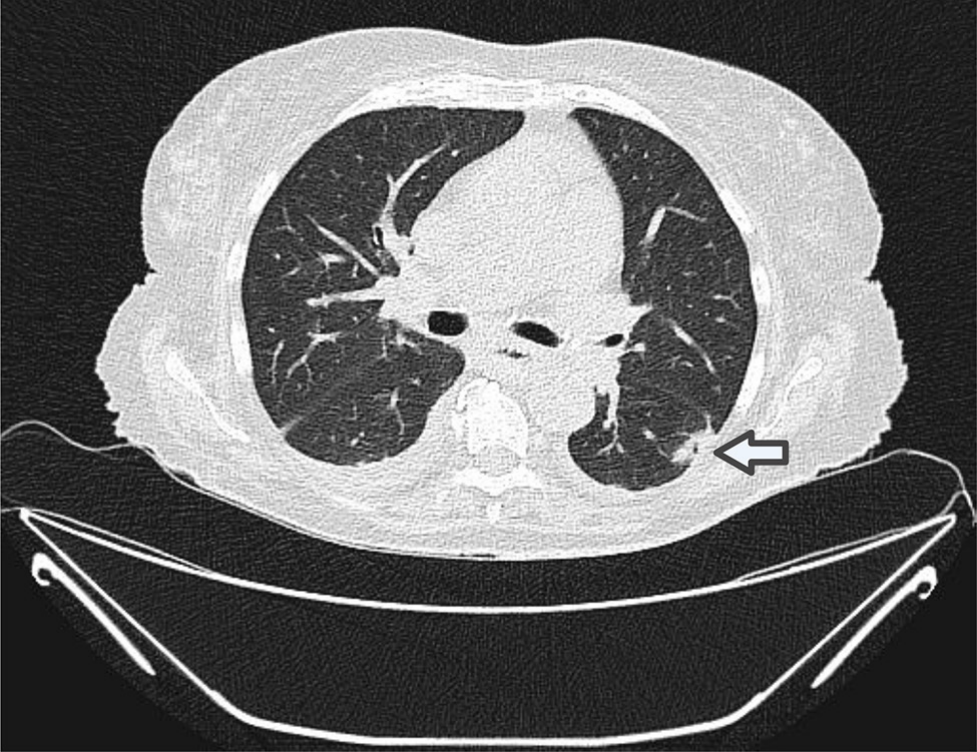
Chest computed tomography scan from an 82-year-old female patient with acute myeloid leukemia in study of *Bjerkandera* spp. pulmonary infection in immunocompromised hosts, Germany. Image depicts nodular infiltrates and a cavitory lesion (arrow) in the left lower lobe suggestive of fungal pneumonia. We cultured and identified a mold from bronchoalveolar lavage fluid as *Bjerkandera* spp. by internal transcribed spacer sequencing.

Patient 2 was an 82-year-old woman who was referred to the University Hospital Cologne with a diagnosis of acute myeloid leukemia 5 months before admission (Table). She had been treated with azacitidine monotherapy, but after allergic transfusion reaction to platelets, her cancer treatment was discontinued. At admission, the patient was experiencing hyperleukocytosis and neutropenia. We initiated cytoreductive treatment with hydroxyurea. A baseline chest CT scan revealed nodular infiltrates and a subpleural cavitary lesion, suggestive of fungal pneumonia (Figure 1). Of note, the patient had not received any antifungal prophylaxis other than trimethoprim/sulfamethoxazole. Bronchoscopy demonstrated purulent secretions. Results of galactomannan testing of BAL fluid were positive; culture yielded a preliminary phenotypic identification of *Geotrichum* spp. All other diagnostic work-up results were unremarkable (Table). Given the clinical significance of the mold identification, we pursued further species-level analysis. ITS sequencing identified the organism as either *B. adusta* or *B. fumosa* (*10*). *Aspergillus* PCR was negative. We could not perform antifungal susceptibility testing because of insufficient fungal growth. We initiated voriconazole therapy but switched to isavuconazole because the patient experienced visual disturbances. Follow-up chest CT scans at 2 and 5 weeks showed stable disease. After 5 weeks of antifungal therapy, we discontinued treatment and initiated posaconazole as secondary prophylaxis. No additional follow-up visits occurred.

**Figure 2:**
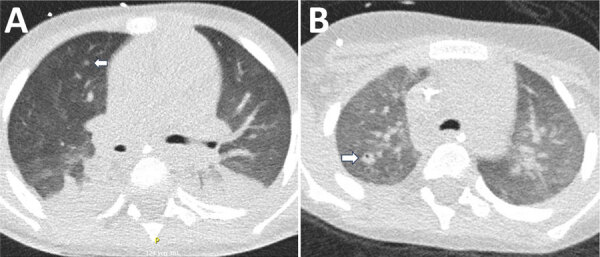
Chest computed tomography scan of a 4-year-old male patient with acute myeloid leukemia in study of *Bjerkandera* spp. pulmonary infection in immunocompromised hosts, Germany. The patient experienced fever unresponsive to antimicrobial treatment. A) Imaging revealed nodular infiltrates and surrounding ground-glass opacities in both lungs (arrow). *Bjerkandera* spp. was identified from tracheal aspiration. B) Follow-up computed tomography scan after 4 weeks demonstrated regressive nodular lesions and the formation of a cavity in the right upper lobe (arrow).

Patient 3 was a 4-year-old boy with newly diagnosed acute myeloid leukemia who initially received cytarabine-based induction chemotherapy (Table). He experienced seizures secondary to cerebral hemorrhage, requiring mechanical ventilation. After successful extubation, we resumed chemotherapy using a high-dose cytarabine and mitoxantrone regimen. He received micafungin (4 mg/kg 2×/wk) as antifungal prophylaxis. Subsequently, he experienced febrile neutropenia (temperature 39.2°C/102.6°F) that was unresponsive to empiric broad-spectrum antimicrobial treatment with meropenem and vancomycin, raising suspicion for IFD. We initiated voriconazole. Serum galactomannan test results were negative. A chest CT scan demonstrated nodular infiltrates and a new cavitary lesion radiographically consistent with a mold infection (Figure 2, panel A). We switched antifungal therapy to liposomal amphotericin B (L-AmB). Fungal culture from a tracheal aspirate yielded a mold with sterile mycelium, which we identified via ITS sequencing as *B. adusta* or *B. fumosa* (*10*). Other diagnostic assessments yielded no findings (Table). After 2 weeks of L-AmB therapy, the patient experienced severe hypokalemia, requiring a switch back to voriconazole. A follow-up CT scan performed 3 weeks later demonstrated a radiographic response to treatment (Figure 2, panel B). No further imaging was done. The patient continued voriconazole therapy for a total of 4 months.

## Conclusions

We described 3 cases with probable invasive lung infections caused by *Bjerkandera* spp. in 2 adults and 1 pediatric patient with hematologic malignancies. We identified no other fungal pathogens or alternative infectious agents by culture, PCR, or serology. All cases met criteria for probable invasive pulmonary mold infection (*11*). Multidisciplinary teams discussed the possibility of contamination and likelihood of invasive disease by *Bjerkandera* spp. and concluded that the identification of *Bjerkandera* spp. was consistent with an IFD in each case, warranting antifungal treatment.

**Figure 3:**
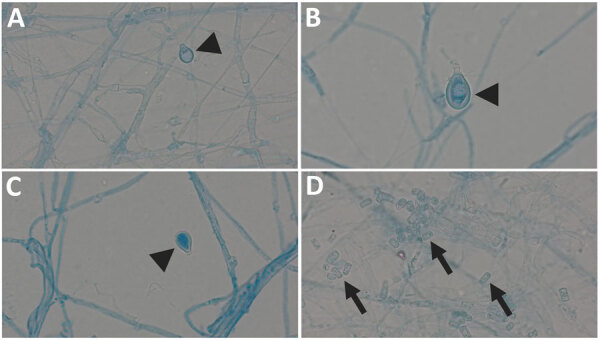
Images of *Bjerkandera* spp. formations from study of *Bjerkandera* spp. pulmonary infection in immunocompromised hosts, Germany. Slides are of lactophenol preparation (original magnification ×1,000). *Bjerkandera* spp. form white, yellowish-white, or tan colonies with a cottony to woolly texture on malt extract agar. The hyphae can be branched. Thin-walled, rectangular arthroconidia are formed via schizolytic dehiscence. In addition, ellipsoidal chlamydospores <10 µm long may develop. Arrowheads indicate chlamydospores (A–C), arrows indicate arthroconidia (D).

We identified the fungus in all 3 cases by sequencing techniques, underlining the importance of molecular approaches in the evaluation of rare fungal infections. In patient 2, *B. adusta* was preliminary identified as *Geotrichum* spp. based on phenotypic appearance. *Geotrichum* spp. are environmental fungi that cause opportunistic infections in at-risk populations (*12*). Both fungi share phenotypic features such as whitish, fluffy to woolly colony morphology and wide-branching septate hyphae with formation of arthroconidia and only occasional formation of chlamydospores (Figure 3) (*6*,*13*). Therefore, reliable identification in filamentous basidiomycetes requires additional techniques such as matrix-assisted laser desorption/ionization time-of-flight mass spectrometry and sequencing methods. Sequence analysis of the ITS ribosomal DNA has better accuracy for species identification; however, for rare fungi, reference data may be incomplete or unavailable for both methods (*14*).

Guidance for clinical management of emerging IFD remains limited because IFD is rare and clinical manifestations vary. Susceptibility test results of 14 *B. adusta* isolates included high MIC for fluconazole and flucytosine and low MIC for AmB and newer triazoles (*15*). Clinical improvement with itraconazole treatment has been described (*14*) in cases with chronic cough associated with *Bjerkandera* spp., consistent with in vitro susceptibility findings. One reported case-patient with invasive rhinosinusitis caused by *Bjerkandera* was treated sequentially with L-AmB, posaconazole, and voriconazole, leading to clinical recovery (*9*). We used newer triazoles and L-AmB for treatment with variable responses. The lack of comprehensive susceptibility testing and outcome data limits definitive treatment recommendations for suspected IFD caused by *Bjerkandera* spp. Describing an unusual pathogen carries a risk for error. We were unable to demonstrate fungal growth in independent respiratory specimens or to obtain histologic proof of invasive growth from lung biopsy.

Our findings suggest that *Bjerkandera* spp. is a human pathogen causing invasive fungal pneumonia or other pulmonary infection in persons at risk, including the immunocompromised. Evaluating the clinical relevance of such infections must consider the degree of immunosuppression and the patient’s future treatment plans. 
